# Explanation and Elaboration with Examples for METRICS (METRICS-E3): an initiative from the EuSoMII Radiomics Auditing Group

**DOI:** 10.1186/s13244-025-02061-y

**Published:** 2025-08-13

**Authors:** Burak Kocak, Angela Ammirabile, Ilaria Ambrosini, Tugba Akinci D’Antonoli, Alessandra Borgheresi, Armando Ugo Cavallo, Roberto Cannella, Gennaro D’Anna, Oliver Díaz, Fabio M. Doniselli, Salvatore Claudio Fanni, Samuele Ghezzo, Kevin B. W. Groot Lipman, Michail E. Klontzas, Andrea Ponsiglione, Arnaldo Stanzione, Matthaios Triantafyllou, Federica Vernuccio, Renato Cuocolo

**Affiliations:** 1 Department of Radiology, Basaksehir Cam and Sakura City Hospital, Istanbul, Turkey; 2 Department of Biomedical Sciences, Humanitas University, Milan, Italy; 3 Department of Diagnostic and Interventional Radiology, IRCCS Humanitas Research Hospital, Milan, Italy; 4 Department of Translational Research, Academic Radiology, University of Pisa, Pisa, Italy; 5 Department of Diagnostic and Interventional Neuroradiology, University Hospital Basel, Basel, Switzerland; 6 Department of Pediatric Radiology, University Children’s Hospital Basel, Basel, Switzerland; 7 Department of Clinical, Special and Dental Sciences, University Politecnica delle Marche, Ancona, Italy; 8 Department of Radiology, University Hospital “Azienda Ospedaliero Universitaria delle Marche”, Ancona, Italy; 9 Division of Radiology, Istituto Dermopatico dell’Immacolata, IRCCS, Rome, Italy; 10 Department of Biomedicine, Neuroscience, and Advanced Diagnostics (Bi.N.D.), University of Palermo, Palermo, Italy; 11 Department of Diagnostic Imaging and Stereotactic Radiosurgery, Centro Diagnostico Italiano S.p.A., Milan, Italy; 12 Departament de Matemàtiques i Informàtica, Universitat de Barcelona, Barcelona, Spain; 13 Computer Vision Center, Bellaterra, Spain; 14 Neuroradiology Unit, Fondazione Istituto Neurologico Carlo Besta, Milano, Italy; 15 Nuclear Medicine Department, IRCCS San Raffaele Scientific Institute, Milan, Italy; 16 Radiomics.bio, Liege, Belgium; 17 Department of Radiology, Netherlands Cancer Institute, Amsterdam, the Netherlands; 18 Department of Thoracic Oncology, Netherlands Cancer Institute, Amsterdam, the Netherlands; 19 Artificial Intelligence and Translational Imaging (ATI) Lab, Department of Radiology, School of Medicine, University of Crete, Heraklion, Greece; 20 Division of Radiology, Department of Clinical Science Intervention and Technology, Karolinska Institute, Stockholm, Sweden; 21 Department of Advanced Biomedical Sciences, University of Naples Federico II, Naples, Italy; 22 Department of Medical Imaging, University Hospital of Heraklion, Crete, Greece; 23 Department of Medicine, Surgery and Dentistry, University of Salerno, Baronissi, Italy

**Keywords:** Radiomics, Artificial intelligence, Machine learning, Quality assessment, Guideline

## Abstract

**Abstract:**

Radiomics research has been hindered by inconsistent and often poor methodological quality, limiting its potential for clinical translation. To address this challenge, the METhodological RadiomICs Score (METRICS) was recently introduced as a tool for systematically assessing study rigor. However, its effective application requires clearer guidance. The METRICS-E3 (Explanation and Elaboration with Examples) resource was developed by the European Society of Medical Imaging Informatics—Radiomics Auditing Group in response. This international initiative provides comprehensive support for users by offering detailed rationales, interpretive guidance, scoring recommendations, and illustrative examples for each METRICS item and condition. Each criterion includes positive examples from peer-reviewed, open-access studies and hypothetical negative examples. In total, the finalized METRICS-E3 includes over 200 examples. The complete resource is publicly available through an interactive website.

**Critical relevance statement:**

METRICS-E3 offers deeper insights into each METRICS item and condition, providing concrete examples with accompanying commentary and recommendations to enhance the evaluation of methodological quality in radiomics research.

**Key Points:**

- As a complementary initiative to METRICS, METRICS-E3 is intended to support stakeholders in evaluating the methodological aspects of radiomics studies.
- In METRICS-E3, each METRICS item and condition is supplemented with interpretive guidance, positive literature-based examples, hypothetical negative examples, and scoring recommendations.
- The complete METRICS-E3 explanation and elaboration resource is accessible at its interactive website.

**Graphical Abstract:**

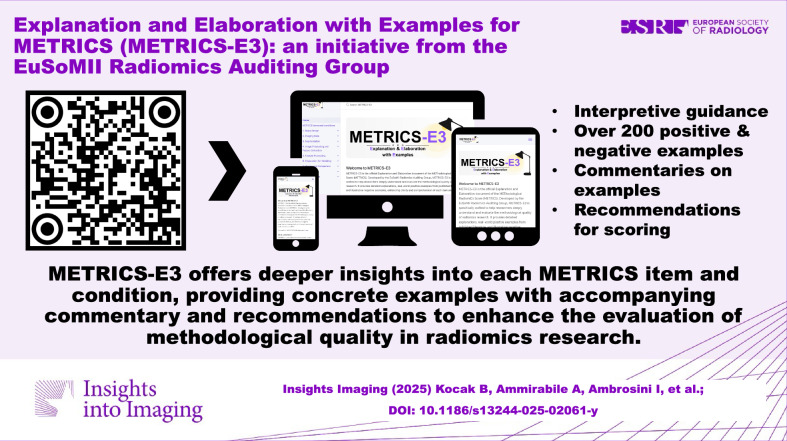

## Introduction

Radiomics refers to the high-throughput extraction and analysis of quantitative features from medical imaging data to identify features that capture underlying pathophysiological processes or phenotypic variations [1]. Its core premise is that medical images contain complex, biologically meaningful information imperceptible to the human eye, but accessible through computational methods. Radiomic analyses, using either hand-crafted or deep learning (DL)-based approaches [2], are increasingly used to develop predictive and prognostic models across various clinical domains, including diagnosis [3, 4], treatment response evaluation [5, 6], genomic correlation or proteomic expression [7–9], and outcome prediction [10, 11].

The field of radiomics has experienced rapid growth over the past decade. As of April 2025, more than 14,000 PubMed-indexed publications include the term “radiomics,” nearly half of which were published since 2023. A recent bibliometric analysis reported an annual publication growth rate of approximately 29% and a short doubling time, reflecting sustained and increasing interest from the research community [12]. Despite this momentum and a proliferation of studies reporting favorable results [13–15], the clinical implementation of radiomics remains limited. A substantial and widening gap persists between the volume of research outputs and their translation into routine clinical practice [16, 17].

This translational gap can be attributed to several factors, particularly methodological complexities. Radiomics involves a multi-step pipeline, including image acquisition, data sampling, segmentation, feature extraction, modeling, and validation, each of which introduces potential sources of bias and variability [18–21]. Heterogeneity in study design and analytical practices, coupled with underpowered studies and inadequately justified sample sizes [22], further compromises reproducibility and generalizability. Moreover, as demonstrated by the two recent coincidental and independent largest umbrella review-style meta-research studies [23, 24], the overall methodological quality of radiomics research remains largely poor and highly inconsistent, posing a significant barrier to its clinical translation.

Recognizing these challenges, recent initiatives have sought to improve standardization and transparency. The Image Biomarker Standardisation Initiative (IBSI) has advanced efforts to harmonize feature extraction protocols [25, 26]. In parallel, consensus-based reporting guidelines such as the Checklist for EvaluAtion of Radiomics research (CLEAR) have been developed to enhance the quality and transparency of study reporting [27]. However, reporting guidelines, while valuable, are not designed to assess methodological rigor and quality of the published research. A study may be transparently reported yet fundamentally flawed in its design or analytical execution.

To address this need and as an alternative to the well-known radiomics quality score (RQS), the METhodological RadiomICs Score (METRICS) was recently developed as a domain-specific quality assessment tool designed to systematically evaluate the methodological rigor of radiomics research [28, 29]. Endorsed by the European Society of Medical Imaging Informatics (EuSoMII), METRICS comprises 30 items across nine categories, covering both handcrafted and DL-based approaches. Each item is weighted according to expert consensus, derived through a modified Delphi process involving an international panel. The tool is condition-specific, reflecting the diversity of radiomics workflows, including traditional hand-crafted pipelines and DL-based approaches, including computer vision. Final scores are calculated on a standardized 0–100% scale via an interactive online platform (https://metricsscore.github.io/metrics/METRICS.html).

Since its introduction in 2024, METRICS has gained rapid uptake, evidenced by several systematic reviews using it [15, 30–47], over 100 citations as of April 2025, and scientific community support [48–50]. Its initial evaluation in controlled settings demonstrated good intra-rater reliability; however, inter-rater reliability was found to be lower, indicating variability in the interpretation and application of specific items [50]. Similar concerns have been echoed in subsequent focused studies assessing METRICS under varying conditions [15]. Moreover, the METRICS framework has been used in studies exploring the use of large language models to automate quality assessment in radiomics research, with findings ranging from poor to moderate and poor to good agreement depending on the tool used [48]. Collectively, these findings underscore the need for enhanced interpretive resources to improve consistency, reproducibility, and usability across diverse evaluative contexts.

To address these gaps, we introduce METRICS-E3 (Explanation and Elaboration with Examples), a companion resource developed to support the consistent and informed use of the METRICS framework in evaluating the methodological quality of radiomics studies. METRICS-E3 offers detailed rationale, interpretive guidance, illustrative examples, and scoring recommendations for each METRICS item. Modeled after similar initiatives such as CLEAR-E3 [51], this resource aims to enhance the interpretability, adoption, and impact of the METRICS tool.

## Development of METRICS-E3

### Contributor recruitment and project Initiation

The METRICS-E3 project was introduced during the 2024 annual scientific project planning session of the EuSoMII Radiomics Auditing Group. Contributors were recruited through an open call within the group. The project was initiated and coordinated by the lead author (B.K.) under the supervision of the senior author (Re.Cu.).

### Task assignment

Contributors were each assigned one or two METRICS items or conditions [28]. Detailed instructions were provided to ensure the selection of diverse, relevant examples aligned with open-access standards. Each contributor was required to collect at least three distinct positive examples per item or condition. Contributors were encouraged to include data from both text and visual content (e.g., tables and figures).

In METRICS-E3, each METRICS item or condition was accompanied by:

- A rationale explaining its importance.
- Positive examples from the literature that demonstrate appropriate adherence to the respective item or condition.
- Hypothetical negative examples illustrating non-adherence, provided for contrast.
- Commentary elaborating on each positive and negative example.
- Scoring guidance to ensure consistent application of METRICS criteria.

### Literature curation for positive examples

Selection of positive examples was based on the alignment (i.e., positive score) with the specific METRICS criterion definition, rather than overall methodological quality of the whole study.

Preference was given to examples sourced from open-access articles, particularly those published under creative commons (CC) licenses, to enable compliant reuse with appropriate attribution. No restriction was applied for specific scholarly databases (e.g., PubMed, Scopus, and Web of Science).

In cases where open-access materials were unavailable, contributors referred to subscription-based articles solely for the purpose of linking to their publicly accessible repositories, without reproducing any cgqORCID="http:righted text, figures, or tables.

All licensing terms were thoroughly reviewed by the lead author to ensure adherence to copyright regulations.

### Generation of hypothetical negative examples

Following internal discussions, the group collectively decided to avoid using real-world negative examples from the literature to prevent ethical concerns related to the identification or critique of individual researchers. Instead, all negative examples were deliberately constructed as hypothetical scenarios designed to illustrate non-compliance with the METRICS criteria, representing common methodological pitfalls that would not meet the criteria. When appropriate, large language models (ChatGPT-4o and Gemini 2.0 Flash) were used to assist in generating and expanding these examples, with various prompts, under the supervision of the lead author (B.K.).

### Example presentation

Positive examples were either quoted verbatim or adapted for clarity, with any omissions clearly indicated using bracketed ellipses (e.g., “[…]”). To minimize potential confusion or misattribution, all citations and references to unrelated figures or tables were intentionally omitted. In cases where figures and tables from the same source were included, their numbering was adjusted to align with the current document’s structure.

All source articles were cited following the examples, with explicit clarification of their CC licenses.

### Internal review and consensus

Once individual contributions were submitted, the lead author (B.K.) conducted a thorough review and revision of all materials to ensure consistency, clarity, and overall quality. This phase was also accompanied by supervision by the last author (Re.Cu.), with several discussions on items and conditions. For each METRICS item or condition, at least two positive and two hypothetical negative examples were selected as representative illustrations.

Additionally, recent findings from the METRICS reproducibility study [50], along with related studies with similar analyses [15], were carefully considered, particularly for items previously shown to exhibit low reproducibility, which corresponds to about half the items, to guide nuanced revisions and improve reliability. These items were handled with particular care during final editing, without a uniform and explicit strategy. Considering potential sources of the reproducibility issues, these items received tailored enhancements, such as expanded recommendations or iteratively refined examples, to improve clarity and support more consistent scoring.

The revised content, comprising both types of examples, was then shared among the full contributor group for collective evaluation and consensus, during which contributors were free to suggest edits or raise concerns on any content. Throughout this process, the senior author (Re.Cu.) again provided ongoing oversight and performed a final comprehensive review to ensure alignment with the project’s objectives.

### Finalized METRICS-E3 and access

The finalized METRICS-E3 includes a total of 227 examples across 30 items and 5 conditions, comprising 124 positive examples sourced from literature and 103 hypothetical negative examples.

To facilitate usability, METRICS-E3 is hosted on an interactive website accessible at: https://radiomic.github.io/METRICS-E3/. The corresponding repository, which also enables version tracking, is publicly available at: https://github.com/radiomic/METRICS-E3.

**Fig. 1:**
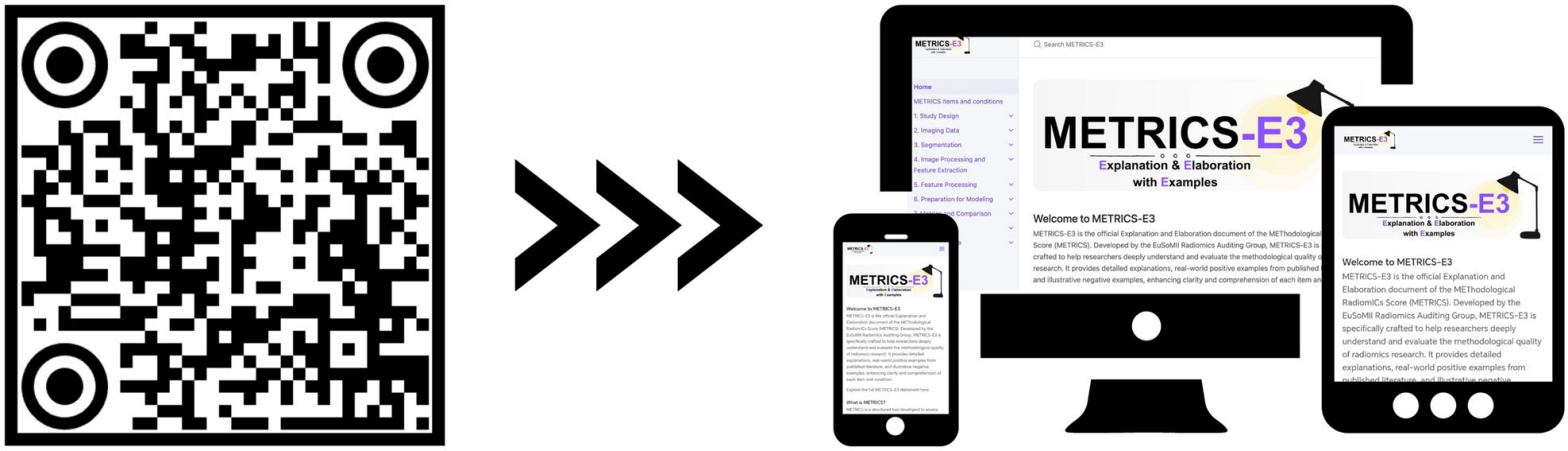
QR code and responsive display of the METRICS-E3 website. Scanning the QR code directs users to the METRICS-E3 web interface (https://radiomic.github.io/METRICS-E3/), which is optimized for use across desktop, tablet, and mobile devices

**Fig. 2:**
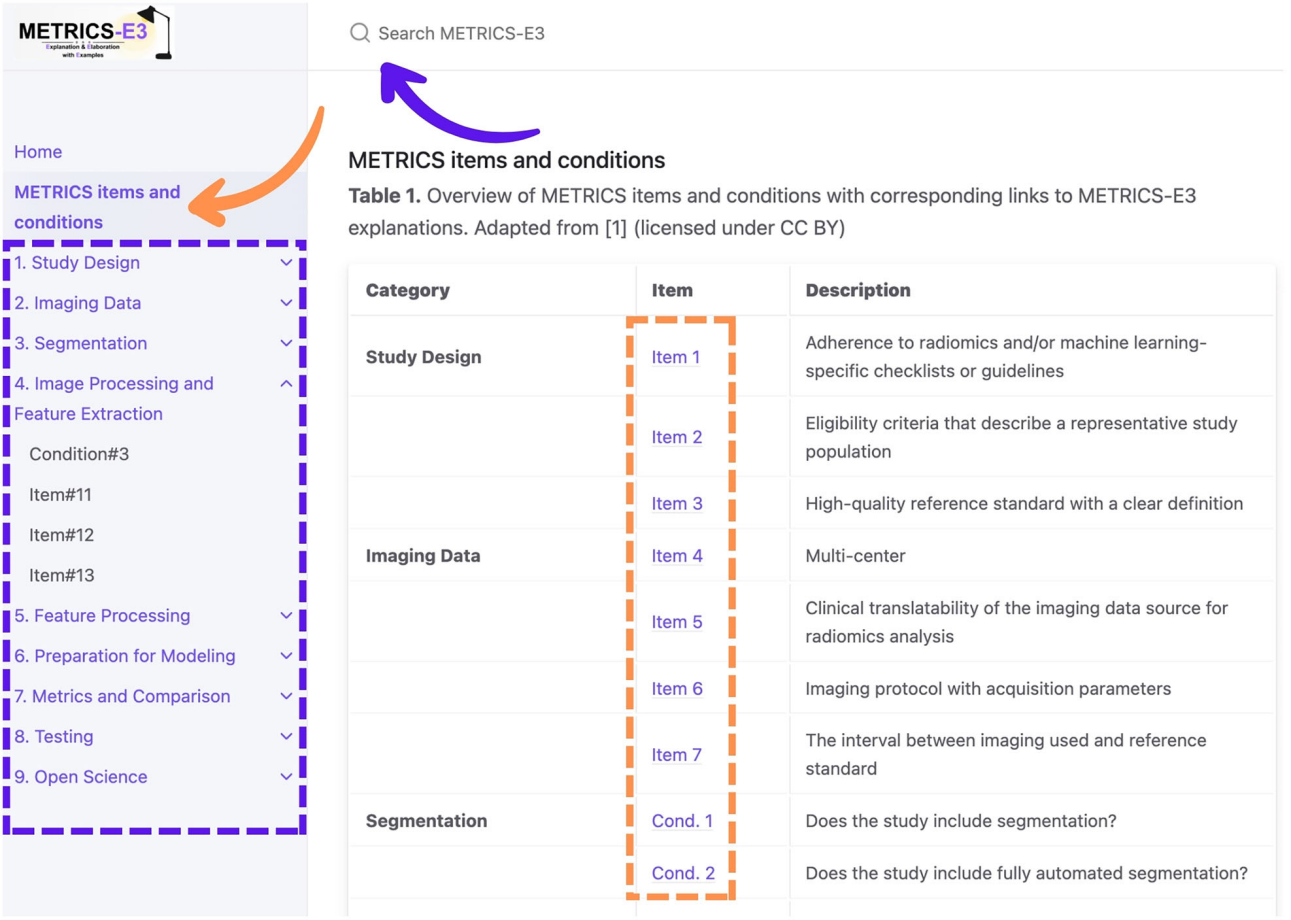
Website functionalities. Users can view all METRICS items and conditions via the item list section (orange rectangular box) and navigate directly to the corresponding content on the “METRICS Items and Conditions” page (orange arrow). Navigation panel (purple rectangular box) includes dropdown menus for accessing specific item or condition pages based on categories. The advanced search function (purple arrow) allows users to quickly locate specific items or search for keywords and concepts within METRICS-E3

**Fig. 3:**
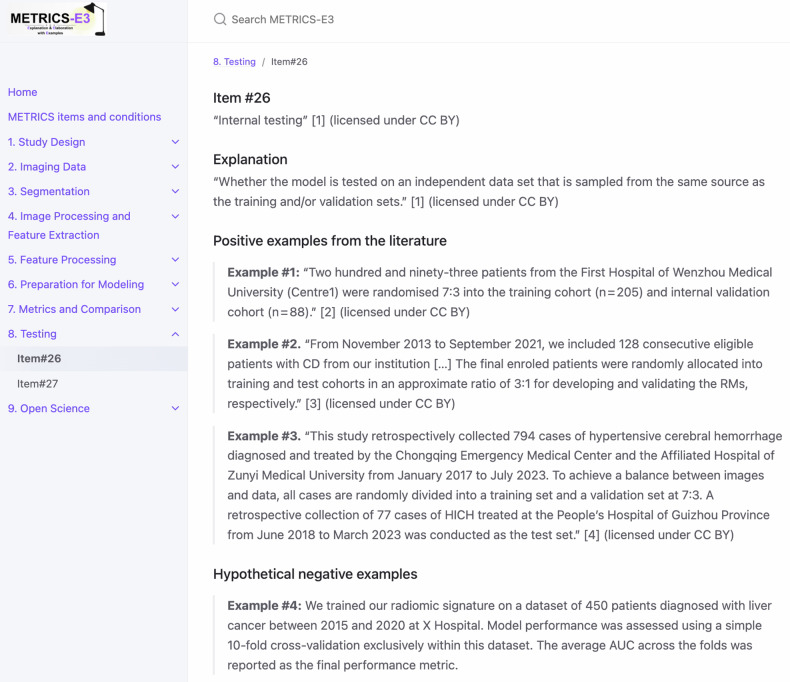
A sample item from METRICS-E3’s interactive website. The entire web page is accessible at https://radiomic.github.io/METRICS-E3/

Figure 1 presents the QR code and responsive display of the METRICS-E3 website. Figure 2 presents the website functionalities. Figure 3 provides a sample item from METRICS-E3.

## Recommendations for using METRICS-E3 in conjunction with the METRICS tool

**Fig. 4:**
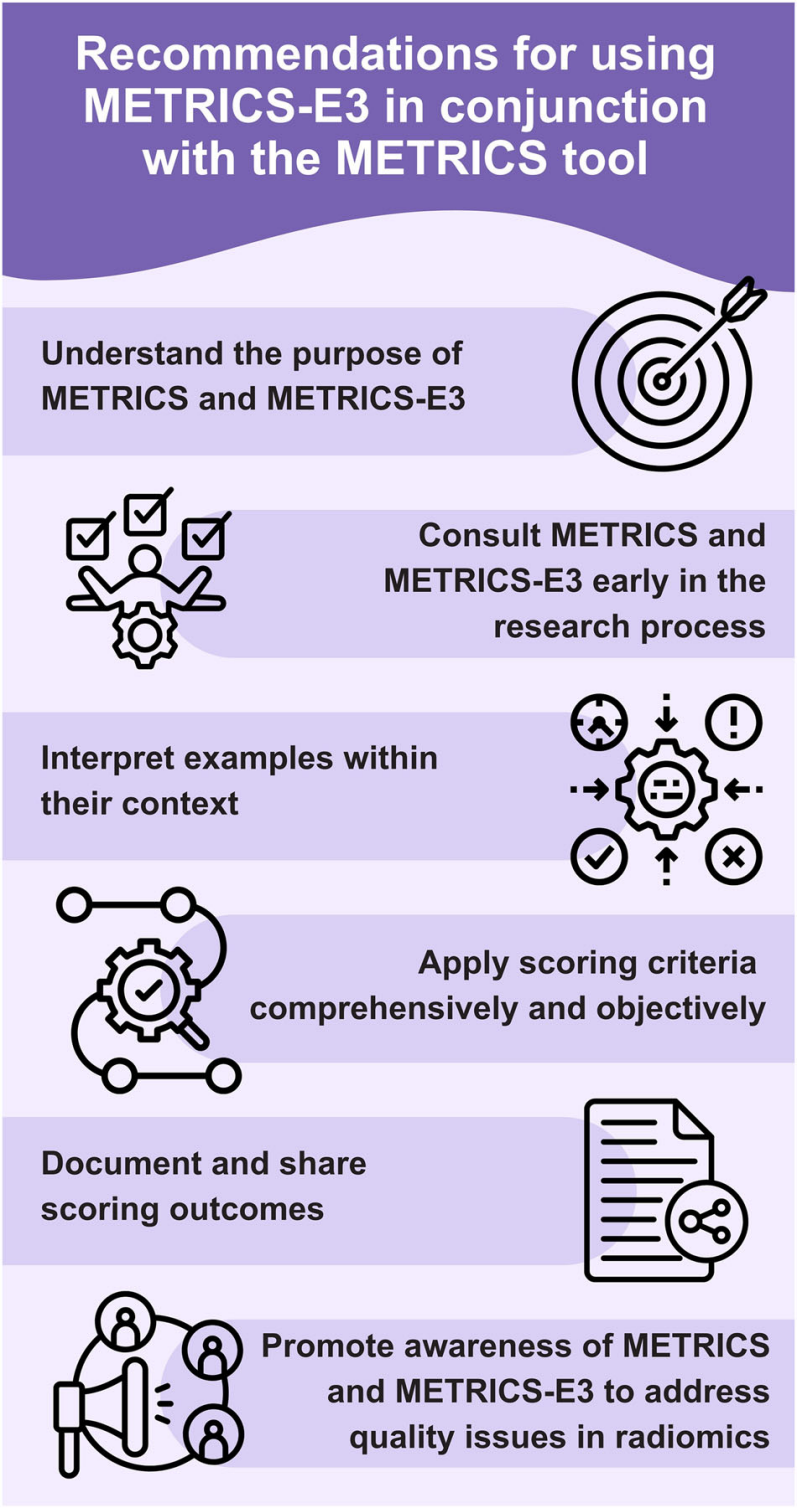
General recommendations for using METRICS-E3 in conjunction with the METRICS tool

The METRICS-E3 working group encourages users of the METRICS tool to consider the following recommendations to ensure proper and effective application of the METRICS quality evaluation framework (Fig. 4). The METRICS tool is available at https://metricsscore.github.io/metrics/METRICS.html.

### Understand the purpose of METRICS and METRICS-E3

METRICS is a structured quality scoring tool designed to evaluate the methodological quality of radiomics studies [28], not to guide manuscript reporting. METRICS-E3 is the official Explanation and Elaboration document, enriched with illustrative examples and commentary. It complements METRICS by offering clarity and guidance on how to interpret and apply each item and condition, thereby supporting consistent and reproducible scoring practices. METRICS-E3 is not a substitute for the METRICS tool but serves as an educational and interpretive resource for researchers, reviewers, and editors.

### Consult METRICS and METRICS-E3 early in the research process

Although METRICS is intended for post hoc quality assessment, researchers planning new radiomics studies may benefit from reviewing it along with METRICS-E3 early on. This approach can help establish methodological standards expected in high-quality research and minimize common design flaws that may affect future evaluability.

### Interpret examples within their context

METRICS-E3 includes a selection of positive examples from published studies and hypothetical negative examples for each item or condition. These examples illustrate how adherence or non-adherence might appear in practice. However, the examples are not exhaustive or prescriptive, and high-quality methodological implementation is not limited to the forms demonstrated. Importantly, the inclusion of a positive example does not imply that the entire study was methodologically sound or scored highly on all METRICS items.

### Apply scoring criteria comprehensively and objectively

Scoring with METRICS should be conducted through a systematic, item-by-item evaluation guided by the definitions and interpretive support provided in METRICS-E3. Each item or condition must be assessed in its entirety, and partial credit should be granted only when explicitly justified by the scoring criteria. METRICS-E3 serves as a valuable resource in this process, both as an educational tool for evaluator training and as a practical reference during the application of the METRICS framework. By providing illustrative positive and negative examples along with detailed recommendations, METRICS-E3 is intended to support consistent and objective application of METRICS criteria; however, its effectiveness in doing so has yet to be formally validated.

For practical purposes, a concise summary of the appropriate scoring recommendations for the five METRICS conditions is presented in Table 1, and for the 30 METRICS items in Table 2. Readers are encouraged to consult the full METRICS-E3 website (https://radiomic.github.io/METRICS-E3/) for comprehensive explanations, examples, and guidance beyond the abbreviated content provided in the tables.

**Table 1:** Summary of the recommendations for the five METRICS conditions

Category	Condition	Definition	Classify as ‘Yes’ if	Classify as ‘No’ if
Segmentation	1	Does the study include segmentation?	• Segmentation is explicitly mentioned and clearly described in the methodology. • Acceptable methods include manual, semi-automatic, or fully automatic segmentation, as well as bounding box or cropping-based approaches. • Visual or textual evidence supports that a region of interest (ROI) was used for analysis.	• No mention of segmentation or use of the entire image without ROI delineation. • Vague or unclear description that does not confirm whether segmentation was performed • Study uses global image features without isolating specific regions.
	2	Does the study include fully automated segmentation?	• Entire segmentation process is automated, with no manual intervention at any stage. • Includes use of deep learning (DL) models without human refinement or correction. • Clearly described workflow confirms absence of manual pre- or post-processing.	• Any manual adjustment or refinement to the automated segmentation. • Semi-automated segmentation involving radiologist corrections. • Manual pre-processing (e.g., ROI selection) or post-processing that influences the final outcome.
Image processing and feature extraction	3	Does the study include hand-crafted feature extraction?	• Radiomics features extracted using predefined mathematical formulas (e.g., GLCM, GLSZM). • Tools like PyRadiomics or LIFEx are used.	• Features extracted using DL models (e.g., CNN layers, autoencoders). • No indication of predefined feature classes or formulas. • Study focusing on deep features extracted in tabular format to be used later on (e.g., for feature selection or machine learning (ML) models).
Feature processing	4	Does the study include tabular data?	• Traditional radiomics features extracted and structured as rows and columns (e.g., using comma-separated or spreadsheet formats). • Deep features extracted and processed separately in tabular format (e.g., for feature selection or ML models).	• DL model used without explicit feature extraction. • No structured numeric representation of features outside the deep network pipeline.
	5	Does the study include end-to-end DL?	• DL model directly maps input data (e.g., images, videos) to output (e.g., prediction, classification) without intermediate explicit feature extraction. • Modular pipelines are allowed if individual components (e.g., segmentation/classification) are themselves end-to-end DL.	• Feature extraction (radiomics or deep features) precedes model training. • DL is only used for feature extraction, followed by traditional ML models (e.g., SVM, logistic regression). • Pipeline includes any intermediary steps between input and final output or disrupts unified DL flow, with the exclusion of modular designs that are end-to-end on their own.

Readers are encouraged to consult the full METRICS-E3 website (https://radiomic.github.io/METRICS-E3/) for comprehensive guidance beyond the abbreviated content provided here. The METRICS tool is available at https://metricsscore.github.io/metrics/METRICS.html

**Table 2:** Summary of the scoring recommendations for 30 METRICS items

Category	Item	Definition	Positive score criteria	Negative score criteria
Study design	1	Adherence to radiomics and/or machine learning-specific checklists or guidelines	• Explicit mention of a radiomics/machine learning-specific checklist (e.g., CLEAR, CLAIM, METRICS).	• No guideline mentioned. • General checklists (e.g., STROBE, STARD) only.
	2	Eligibility criteria that describe a representative study population	• Comprehensive, transparent criteria that reflect the target population.	• Vague or undefined criteria (e.g., “poor image quality,” “missing data”). • Overly restrictive criteria that reduce generalizability (e.g., narrow age range, exclusion of common comorbidities). • Exclusion of typical or prevalent cases that distort clinical representativeness.
	3	High-quality reference standard with a clear definition	• A reference standard is clinically validated and widely accepted (e.g., histopathology, consensus clinical criteria). • Clear, detailed definition of how the reference standard was applied in the study. • Applied consistently across all cases, with justification for its use.	• Subjective reference standard (e.g., radiologist’s opinion without follow-up or histopathology). • Vague or no definition provided for the reference standard. • Inconsistently applied or lacks justification for choice and implementation.
Imaging data	4	Multi-center	• Data collected from two or more independent institutions with differing patient populations and imaging protocols. • Institutions are not affiliated or do not share identical scanners/protocols. • Study explicitly names the centers and describes their distinct characteristics.	• Data from a single institution only, regardless of internal data split. • Multiple centers from the same healthcare network with similar protocols/scanners. • Misleading use of terms like “external validation” for internal or single-site splits. • Studies do not explicitly name the centers.
	5	Clinical translatability of the imaging data source for radiomics analysis	• Explicit use of standardized imaging protocols (e.g., PI-RADS, BI-RADS, Lung-RADS). • Citation of relevant guidelines or publications validating the protocol.	• No mention of standardized protocols or adherence to guidelines. • Use of heterogeneous, experimental, or site-specific imaging protocols without justification. • Reliance on local or undocumented acquisition settings that limit reproducibility.
	6	Imaging protocol with acquisition parameters	• Detailed reporting of scanner type(s) and all relevant acquisition parameters (e.g., slice thickness, kVp, TR/TE, *b*-values). • Protocol details are specified separately for training and testing datasets.	• Vague or qualitative protocol descriptions (e.g., “standard protocol,” “approximately 5 mm” slice thickness). • Missing key acquisition parameters (e.g., field strength, reconstruction kernel, contrast use). • No mention of scanner models or parameter variation across datasets.
	7	The interval between the imaging used and the reference standard	• Clearly defined and clinically justified time interval between imaging and outcome/reference standard. • Short interval when diagnostic accuracy is critical (e.g., < 2 weeks for diagnosis-related studies). • Interval appropriate to the study aim (e.g., long-term follow-up justified in prognostic models). • For segmentation studies, the interval can be assumed to be “zero”.	• No mention or unclear timing between imaging and outcome/reference standard. • Time interval is likely to introduce bias due to disease progression or treatment effects. • Lack of rationale for chosen interval, especially when extended periods may impact data validity.
Segmentation	8	Transparent description of segmentation methodology	• Clear specification of segmentation tool/software, method (manual, semi-automatic, automatic), and number of readers. • Detailed description of the image type, orientation used for segmentation, as well as slice selection methodology in case of 2D segmentation. • For peri-tumoral regions or cropping: defined size, method, and rationale, possibly illustrated with figures.	• No mention of the segmentation tool, method, or who performed it. • Missing details about the image sequence or orientation used for segmentation. • Segmentation or cropping details (e.g., size, slice selection) omitted or vaguely described.
	9	Formal evaluation of fully automated segmentation	• Quantitative assessment reported (e.g., Dice similarity coefficient, Jaccard index) comparing automated segmentation to manual ground truth. • Transparent description of segmentation tool, evaluation methodology, and reference annotations.	• No quantitative evaluation of segmentation accuracy (e.g., metrics not reported). • Misclassified as fully automated while using manual correction or radiologist adjustments (i.e., semi-automated). • Reliance on commercial or pre-trained models without validation against ground truth.
	10	Test set segmentation masks produced by a single reader or an automated tool	• Test set region of interest segmented by a single radiologist or a fully automated method. • Manual corrections by only one reader in semi-automated workflows. • Reproducibility analyses with multiple readers limited to the training set only.	• Test set segmentation involves multiple readers or consensus adjustments. • Manual corrections performed by more than one reader, even if the initial segmentation was automated. • No clear statement on who segmented the test set or the segmentation method used in the test set.
Image processing and feature extraction	11	Appropriate use of image preprocessing techniques with transparent description	• For traditional radiomics (at least): detailed reporting of resampling (including voxel size), normalization, and intensity discretization. • For DL (at least and if applicable): description of resizing, normalization method, and resampling. • Tailored preprocessing methods included for specific modalities (e.g., bias field correction for MRI).	• Missing or vague details on key preprocessing steps (e.g., unspecified voxel size, method of normalization). • Reference to default software settings without specifying software/version or method.
	12	Use of standardized feature extraction software	• Use of IBSI-compliant software with reference to IBSI guidelines or documentation, with software name and version clearly reported.	• In-house or non-validated scripts used without evidence of standardization. • Standardized tool mentioned, but version or documentation missing (e.g., “PyRadiomics” without version)
	13	Transparent reporting of feature extraction parameters, otherwise providing a default configuration statement	• Complete configuration reported (e.g., via yaml file, script, and full parameter list). • Explicit confirmation that non-reported parameters were kept at default settings. • For DL: full architecture and preprocessing pipeline described from input to output.	• Use of commercial or unnamed software without configuration transparency. • No confirmation statement that non-reported parameters were kept at default settings. • Missing or vague description of DL model structure, preprocessing, or hyperparameters.
Feature processing	14	Removal of non-robust features	• Explicit use of reproducibility/stability testing methods (e.g., test-retest, inter-reader analysis for segmentation-based feature reproducibility, perturbation testing).	• No assessment of feature variability due to scanner, segmentation, or acquisition changes. • Focus only on removing redundant or collinear features without considering robustness.
	15	Removal of redundant features	• Explicit removal of highly correlated, redundant, non-informative features (e.g., using correlation analysis, L1 regularization, feature selection algorithms).	• Methods used do not eliminate redundancy of individual features (e.g., principal component analysis, clustering). • Focus only on missing data, variability, or robustness without addressing feature correlation. • Redundancy reduction is implied but not explicitly described or executed.
	16	Appropriateness of dimensionality compared to data size	• Dimensionality justified using an appropriate method (e.g., Riley’s approach). • Assessment of model fit using uncertainty estimates (e.g., overlapping 95% confidence intervals for AUC between training and testing). • Number of features proportionate to the number of patients in both the total and minority classes, justified using a valid analytical approach.	• Too many features relative to sample size or minority class without justification using a valid analytical approach. • Performance drop between training and testing cohorts with no explanation or uncertainty evaluation. • Sample size justified solely based on metrics like AUC or statistical power without accounting for dimensionality.
	17	Robustness assessment of end-to-end DL pipelines	• Explicit robustness testing (e.g., test-retest reproducibility, inter-reader variability, adversarial attack methods). • Dataset modifications (e.g., cropping, perturbations) applied to simulate real-world variation. • Quantitative evaluation of robustness using appropriate metrics.	• Variability assessed only via retraining with different random seeds. • Use of data augmentation alone without post-training robustness evaluation. • External validation reported without specific tests for robustness under data perturbation or reproducibility scenarios.
Preparation for modeling	18	Proper data partitioning process	• Data split performed before any preprocessing or feature selection steps. • All leakage-prone steps (e.g., feature selection, scaling, and oversampling) are restricted to the training set. • Patient-level splitting is applied to avoid cross-set data leakage and ensure independence.	• Preprocessing (e.g., imputation, scaling, oversampling) is applied before the data split or across all data. • Data split performed at scan level, allowing patient-level leakage. • Feature selection, model tuning, or image preprocessing conducted before or without a clear data split
	19	Handling of confounding factors	• Explicit analysis of known confounders (e.g., age, gender, tumor size, acquisition protocol). • Statistical correction applied where confounders are identified, with appropriate multiple testing correction. • Use of ablation studies or subgroup analyses to assess confounder influence.	• Clinical variables were added to the model without testing for confounding effects. • Confounders mentioned post hoc without correction or exclusion. • Only partial/confined analysis (e.g., testing one confounder without evaluating others) or no confounder analysis at all.
Metrics and comparison	20	Use of appropriate performance evaluation metrics for the task	• Task-appropriate metrics reported (e.g., at least: AUC, sensitivity, specificity for classification; MAE/MSE for regression). • Confusion matrix provided for classification tasks. • Loss curves presented for DL models to assess training behavior.	• No confusion matrix for classification tasks. • Missing key metrics (e.g., F1-score reported but sensitivity/specificity not reported in medical classification). • DL models presented without loss curves, preventing assessment of convergence or overfitting.
	21	Consideration of uncertainty	• Uncertainty metrics reported (e.g., 95% confidence intervals, standard deviations, or standard errors). • Validation method used to derive uncertainty (e.g., bootstrapping, k-fold cross-validation, nested cross-validation) is clearly described. • Results presented with variability estimates for key performance metrics across data splits or subgroups.	• Only point estimates are reported without any uncertainty measures. • Uncertainty measures (e.g., confidence interval, standard deviation) are included but without methodological explanation for deriving these measures (e.g., validation method or resampling approach).
	22	Calibration assessment	• Calibration assessed using quantitative metrics (e.g., Brier score, Spiegelhalter’s z-test) or visual plots (e.g., calibration curves). • Calibration reported for at least the test set; ideally also for training and/or validation sets. • Calibration results supported with statistical values or plots (e.g., proximity to 45° line).	• No calibration analysis performed; only discrimination metrics reported. • Calibration is mentioned without providing quantitative results, plots, or test statistics.
	23	Use of uni-parametric imaging or proof of its inferiority	• Features extracted from a single imaging set with clear justification (e.g., PET-only, CT-only). • For multi-parametric/multi-modal studies, uni-parametric models are also evaluated and compared. • Formal statistical tests (e.g., DeLong’s, McNemar’s) used to justify the added value of combined models.	• Multi-modal features combined without uni-parametric (i.e., single modality) comparisons. • Performance of the combined model reported without statistical validation against simpler models with single modality.
	24	Comparison with a non-radiomic approach or proof of added clinical value	• Standard non-radiomic benchmarks (e.g., PI-RADS, LI-RADS, radiologists’ visual interpretation) included in analysis for comparison. • Radiomics-only model compared to clinical or visual assessment models using formal statistical methods (e.g., DeLong’s test, decision curve analysis). • Combined models compared to standalone clinical models with proper statistical evaluation.	• No comparison made to clinical or radiological baseline. • Only qualitative or informal comparisons (e.g., “better than literature”) without dataset-specific analysis. • Performance of models shown side-by-side without statistical testing of differences.
	25	Comparison with simple or classical statistical models	• Complex model compared with a simple/classical model (e.g., logistic regression, no-information rate). • Formal statistical testing applied to evaluate performance differences (e.g., DeLong’s test, net reclassification index). • Clear justification provided for the use of more complex modeling techniques.	• Only complex models compared with each other (e.g., CNN vs ResNet) without a classical baseline. • Simple model included, but no statistical test applied to validate performance difference. • Claimed superiority based on point estimates without formal evaluation against a baseline.
Testing	26	Internal testing	• An independent holdout set drawn from the same population as the training set is used for testing (i.e., internal test set). • Terminology may vary (“validation” or “test”), but data source consistency is key. • Nested cross-validation used with a dedicated outer test fold from the same source population.	• Only a simple cross-validation was performed without a separate holdout set. • Test set derived from a different institution or population (i.e., external testing). • Terminology misuse leads to confusion, and data origin suggests test data is not from the same cohort as the training set.
	27	External testing	• Model tested on a dataset from an institution entirely independent of the training set. • Institutional source of external test set clearly identified and distinguished from training/validation sites.	• All data from a single institution, even if temporally split or mislabeled as “external validation”. • Training and testing sets created by random splitting of pooled multi-center data, without site separation. • No explicit confirmation of an independent institutional source for test data.
Open science	28	Data availability	• Clinical, radiological, segmentation, or radiomics feature data publicly available in repositories (e.g., TCIA) or any others. • The dataset includes sufficient documentation, labeling (e.g., outcome classes), and is directly accessible via link or DOI. • Shared data allows replication or reanalysis (e.g., radiomic features with class labels).	• Data “available upon request” or restricted through approval processes. • Public link missing or dataset stored in non-accessible repositories (e.g., protected by passwords or author-mediated release). • Shared feature values without corresponding class/outcome labels or documentation are insufficient for reuse.
	29	Code availability	• Code publicly available via accessible repositories (e.g., GitHub), with working links. • Includes full implementation (e.g., feature extraction, modeling) with sufficient documentation and comments.	• Code available only “upon request” or behind agreements with the authors. • No code sharing information or broken/non-functional repository links. • Shared code lacks essential components or documentation for reuse and reproducibility.
	30	Model availability	• Final model shared in a usable format (e.g., .pkl, .h5) including learned weights. • Clear documentation or instructions for use (e.g., input format, preprocessing steps, required libraries). • Alternatively, full formulas, coefficients, or a functional nomogram provided with calculation guidance.	• Only training code provided, without final trained model. • Model structure/formula shown but missing essential weights or intercepts. • Shared model file lacks usage instructions, required dependencies, or input data format. • Nomograms shown without Rad-score formula or guidance for prediction by an external user.

Readers are encouraged to consult the full METRICS-E3 website (https://radiomic.github.io/METRICS-E3/) for comprehensive guidance beyond the abbreviated content provided here. The METRICS tool is available at https://metricsscore.github.io/metrics/METRICS.html

### Document and share scoring outcomes

When applying METRICS to assess radiomics studies, such as in systematic reviews, methodological evaluations, or meta-research, researchers are encouraged to include completed METRICS scoring forms or structured summaries as supplementary material or in publicly accessible repositories. Transparent documentation enhances reproducibility, facilitates critical appraisal, and supports data reuse. It also enables large-scale umbrella reviews evaluating different quality aspects across diverse study contexts, regardless of disease type, imaging modality, or other variables [23, 24, 52].

Primary study authors may also use METRICS for self-assessment and share their scoring results alongside manuscripts [53, 54]. This can offer reviewers and editors a concise overview of methodological rigor, helping to streamline the peer review process, which is already under strain [16, 55]. Additionally, this practice supports future meta-research, including analyses of reporting trends and the reliability of self-assessed quality [56]. However, researchers should apply these practices carefully, as prior studies have shown suboptimal implementation of similar approaches for reporting tools [53, 56, 57].

### Promote awareness of METRICS and METRICS-E3 to address quality issues in radiomics

Given the well-documented methodological shortcomings in radiomics research and the persistent gap in clinical translation, promoting the appropriate use of quality assessment tools is essential. Recent meta-research has highlighted the limited adoption of methodological evaluation tools within radiomics [53]. To support the widespread and consistent application of METRICS and its elaboration document, METRICS-E3, we recommend that users reference these in their publications. Reviewers should also assess whether authors appropriately cite and apply METRICS when claiming high methodological quality. In such cases, authors are encouraged to include a standardized statement, such as: “The methodological quality of this study was assessed using the METRICS tool under METRICS-E3 guidance.”

## Challenges encountered during the development of METRICS-E3

During the development of METRICS-E3, several implementation challenges were encountered. First, some METRICS items were inherently broad or complex, requiring nuanced interpretation and iterative clarification as the tool itself evolved. Second, contributors occasionally diverged in their understanding of item intent, necessitating harmonization through centralized review and supervision. Third, ethical concerns prevented the use of real negative examples, requiring the creation of plausible but hypothetical scenarios, which had to be both realistic and aligned with scoring logic. Fourth, licensing restrictions further limited the pool of eligible positive examples to open-access sources with compliant reuse rights. Fifth, maintaining consistency in tone, structure, and adherence to METRICS criteria across a diverse group of contributors added editorial complexity. Throughout, utmost care was taken to ensure that illustrative examples did not unintentionally misrepresent or overextend the intent of METRICS scoring guidance, preserving fidelity to the original tool while improving its interpretability.

## Limitations

Despite its potential educational value, this work has several limitations that could be addressed in future iterations to improve its utility and impact. First, the development of METRICS-E3 involved many contributors who were also involved in the original METRICS tool, potentially introducing homogeneity of interpretation. While this ensured consistency with the original framework, it may limit the generalizability of the guidance without external validation. Second, although considerable effort was made to ensure the plausibility and relevance of illustrative examples, negative examples were necessarily hypothetical due to ethical constraints, and their real-world fidelity has not yet been empirically assessed. Third, the current version lacks formal validation studies, such as inter-rater agreement testing or end-user evaluations (e.g., impact on evaluator training), to quantify its impact on scoring consistency or methodological rigor. Furthermore, METRICS-E3 may serve as a reference resource to support the development and refinement of automated quality assessment tools, including those using large language models [48, 58, 59], which need to be formally assessed as well. All these aspects are planned for future research. Fourth, practical constraints such as open-access licensing requirements limited the selection of literature examples. Otherwise, there may be better examples in subscription-based articles. Fifthly, we did not formally assess selection bias, as the primary criterion was that examples be plausible, clearly aligned with the METRICS item, and educationally valuable. However, we acknowledge that many contributors sourced examples from high-quality, open-access journals, often including ESR-affiliated publications, due to accessibility and licensing considerations. This, along with potential contributor bias toward easily retrievable examples, may introduce some selection bias. Lastly, while METRICS-E3 was developed through structured group review and discussion, it was not based on a formal consensus method such as Delphi, which is uncommon for explanation and elaboration documents but may be beneficial in future elaboration efforts.

## Final remarks

The successful integration of radiomics into clinical practice relies not only on technological advancements but also on the consistent application of rigorous methodological standards. To support this, the METRICS framework was developed as a structured, consensus-based tool for evaluating the quality of radiomics studies [28].

To facilitate its effective use, the METRICS-E3 tool was introduced as a companion guide, offering detailed explanations, scoring suggestions, and practical examples for each item in the framework. Informed by our prior experience with the CLEAR-E3 project [51], METRICS-E3 features a more structured web platform. Key additions include hypothetical negative examples, specific commentaries discussing all the examples, and targeted recommendations for accurate and consistent scoring, helping users distinguish between strong and weak methodological practices according to METRICS.

METRICS-E3 is a collaborative initiative led by the EuSoMII Radiomics Auditing Group. The group remains committed to advancing transparency and quality in radiomics research by launching targeted initiatives with impactful publications and tools. Community feedback is encouraged to ensure that METRICS and METRICS-E3 continue to evolve and support high-quality, clinically relevant research.

## Data Availability

All data is presented in the publication and at https://radiomic.github.io/METRICS-E3/.
